# Tuberculosis infection control practices and associated factors among healthcare workers in hospitals of Gamo Gofa Zone, Southern Ethiopia, institution-based cross-sectional study

**DOI:** 10.1371/journal.pone.0239159

**Published:** 2020-09-21

**Authors:** Addisu Walelign Tadesse, Amisalu Alagaw, Mekidim Kassa, Muluken Bekele

**Affiliations:** 1 Department of Public Health, College of Medicine and Health Science, Selale University, Fiche, Ethiopia; 2 Department of Public Health, College of Medicine and Health Science, Arba Minch University, Arba Minch, Ethiopia; The University of Georgia, UNITED STATES

## Abstract

**Background:**

Tuberculosis infection control is a combination of measures designed to minimize the risk of tuberculosis transmission within populations. Healthcare workers are not sufficiently protected from tuberculosis infection in healthcare facilities where infection control protocols are not followed completely. Studies conducted in Ethiopia about tuberculosis infection control practices were self-report.

**Objective:**

To assess tuberculosis infection control practices and associated factors among health care workers in hospitals of Gamo Gofa Zone, Southern Ethiopia.

**Method:**

A facility-based cross-sectional study was conducted from March 6 to April 2, 2019. The sample size was 422. The sample was proportionally allocated to each hospital and the respective discipline. Simple random sampling was used to select participants from each discipline. Data were entered into EpiData version 4.4.2.1 and analyzed using SPSS Version 21 software. Multicollinearity and Model goodness-of-fit was checked. A multivariate logistic regression model at 95% CI was used to identify the predictors.

**Result:**

The response rate was 97.4%. The proportion of good tuberculosis infection control practice was 39.9% [95% CI (35.5, 44.9)]. Knowledge on tuberculosis infection control measures [AOR = 3.65, 95% CI (2.07, 6.43)], educational level of degree and above [AOR = 2.78, 95% CI (1.7, 4.53)] and ever having tuberculosis-related training [AOR = 2.02, 95% CI (1.24, 3.31)] were significantly associated with good tuberculosis infection control practice.

**Conclusion and recommendation:**

The proportion of good tuberculosis infection control practice among healthcare workers in hospitals of the Gamo Gofa Zone was 39.9%. The good practice of tuberculosis infection control was determined by educational level, working department, knowledge on tuberculosis infection control measures, and having tuberculosis-related training. Hence, training of healthcare workers, targeting diploma-holders in upgrading educational level programs, developing knowledge on tuberculosis infection control measures, and qualitative research to explore reasons for not practicing infection control measures is recommended.

## Introduction

Tuberculosis infection control (TBIC) is a combination of measures designed to minimize the risk of tuberculosis (TB) transmission within populations. It is a subcomponent of the World Health Organization (WHO) updated Stop TB strategy contributing to strengthening of health systems. The foundations for TBIC are early and rapid diagnoses and proper management of TB patients. Hence, TBIC activities are divided into administrative, environmental, and personal protective equipment [1]. The globe had been performing efforts to overcome the problem of TB with STOP-TB strategy up to the end of the Millennium Development Goals era and followed by END-TB strategy following the initiation of Sustainable Development Goals with the target of 95% reduction in the number of TB death and 90% reduction in TB incidence rate by 2035. Healthcare workers (HCWs) are not sufficiently protected from TB infection in health-care facilities where infection control protocols are not followed completely [2].

Tuberculosis is vastly contagious in resource-limited healthcare facilities and HCWs are at increased risk for acquiring TB in such settings [3]. Many studies have shown higher rates of tuberculin skin test conversion [4], M/XDR-TB diagnosis and admittance compared with the general population [5] and some have been shown to have likely been transmitted in a healthcare setting [5].

Healthcare-associated infections are important sources for a substantial burden of diseases among HCWs and patients. World Health Organization reported that the incidence of latent tuberculosis infection (LTBI) and TB disease among HCWs in healthcare facilities exceeds the incidence among the general population or HCWs not exposed to health-care facilities [2]. A systemic review showed that the prevalence of LTBI among HCWs was 37%, the mean incidence rate of TB disease was 97/100000 per year, and the incidence rate ratio for active TB among HCWs compared with the general population was 2.94 [6]. The estimated annual incidence of TB disease among HCWs was 67 cases/100,000 persons, 91/100,000 persons, and 1,180/100,000 persons for studies from countries with low, intermediate, and high TB incidence respectively. The corresponding TB incidence for the general population adopted as comparisons were 33/100,000 persons, 82/100,000 persons, and 311/100,000 persons respectively [7].

Tuberculosis is a major concern of HCWs in sub-Saharan Africa. The median prevalence LTBI in HCWs was 62% and 387/100,000 was the median incidence of TB disease in Sub-Saharan Africa [8]. A finding from Rwanda showed that the prevalence of LTBI was 69% among HCWs compared to school workers (39%) [9]. The prevalence of LTBI was 60% among HCWs compared to school workers (48.2%) in Kenya [10].

The estimated incidence of MDR-TB (multi-drug resistant tuberculosis) hospitalization among HCWs was 64.8/100,000 and 7.2/100,000 for XDR-TB (extensively drug-resistant tuberculosis) hospitalization. The risk of hospitalization due to MDR-TB was 11.9/100,000, and 1.1/100,000 for XDR-TB in the general population [11].

Healthcare workers had fears associated with the risk of acquiring TB, especially drug-resistant TB (DR-TB). Some of them are developing MDR- and XDR-TB, the treatment course, the financial implications, family concerns, working environment, and psychosocial issues; which directly affect the provision of quality health service [12]. Based on a study conducted in South Africa, nearly 50% of the participants felt that the hospital didn’t care about them and/or was not working to prevent staff TB infections, and 42.9% were less motivated to continue as a HCW because of staff TB/MDR-TB and XDR-TB deaths [5].

The magnitude of TBIC practice in Ethiopia ranged from 38% among HCWs in West Gojjam [13] to 63.2% in Northwest Ethiopia [14]. A recent study conducted among HCWs at the University of Gondar Referral Hospital and Felege Hiwot Referral Hospital revealed that self-reported practice on MDR-TB prevention and control was 19.6% [15].

Ethiopia is one of high TB-burdened countries and TB remains a challenging problem even though the Ethiopian government had been working over the past decades to enhance TBIC activities. Despite the fact that guidelines were developed in global and national levels and training has been provided to HCWs, the TBIC practice was not assessed in the study area.

Only a few studies in Ethiopia illustrated HCWs' self-report practice towards TBIC although HCWs are at higher risk of TB and they have a higher role on infection control. Most of the studies about TBIC practice revealed that self-report TBIC practice assessment as a limitation of their findings. Even though most of HCWs had knowledge about TBIC practice they didn’t practice it during observation [16–18]. Therefore, the purpose of this study was to assess TBIC practice among HCWs and to identify factors associated with TBIC practice in Public Hospitals of Gamo Gofa Zone, Southern Ethiopia.

## Methods and materials

### Study area, study period and study design

An institution-based cross-sectional study was conducted from March 6 to April 2, 2019, at public hospitals of Gamo Gofa Zone, Southern Ethiopia. Gamo Gofa Zone, whose capital city is Arbaminch, located at 505 km far from Addis Ababa with an area of 12,581.4 km^2^. The zone is structured in 2 town administrations, 15 woredas, 34 urban kebeles, and 400 rural kebeles. There are 2 general hospitals, 5 primary hospitals, 77 health centers, and 471 health posts in the zone giving services for about 2,040,923 populations. A total of 3,151 health professionals are providing healthcare services in the zone; out of which 913 has been working at the six hospitals. (*Source*: *Gamo Gofa Zone Health Office*, *Planning Department*; *January 2019*).

### Study population

- All HCWs working in all hospitals of the Gamo Gofa Zone were the source population. Health professionals who were working in all public hospitals of Gamo Gofa Zone and qualified as a doctor, health officer, nurses, x-ray technician, pharmacy personnel and laboratory personnel were study populations.

### Inclusion criteria

- Healthcare workers, who were working at Triage, medical adult-OPD, TB-clinics, Emergency, Laboratory, Pharmacy, Radiographic Units, Intensive Care Units, and Internal Medicine wards were included in the study.

### Sample size and sampling procedures

#### Sample size

The sample size was determined as follows based on a single population proportion formula with a 5% marginal error and 48.6% TBIC practice from the previous study [19].

$$\begin{array}{cc} \text{Sample}\;\text{size}=\text{n} & =\frac{\frac{x_{\alpha }}{2}}{d^{2}}p(1-p)\\ & =\frac{1.96^{2}}{0.05^{2}}*0.486(1-0.486)=384; \end{array}$$

considering 10% non-response rate, the final sample size by using a single population proportion formula was 422.

The sample size for factors was determined by using EPI INFO Version 7 Stat calc by considering having TB training, know the presence of TBIC plan and know the presence of national guideline for TBIC from previous studies [13, 19] (S1 Table). Hence due to cost feasibility, the sample size obtained by a single population proportion formula (422) had been taken as the final sample size.

#### Sampling procedures

Proportional numbers of study participants were allocated to each hospital according to their contribution to the total sample size and each discipline. Individual participants who fulfilled the inclusion criteria were selected by simple random sampling from each discipline in each hospital, taking the contribution of each discipline to the sample size into consideration. The total numbers of HCWs (not including midwives) in the six hospitals were 608 (S1 Fig). The sampling fraction was obtained by dividing the sample size (422) by the total number of HCWs (608). This sampling fraction used to allocate the sample size to each hospital was 0.694 [422/608] proportionally.

### Variables of the study

The dependent variable was the TBIC practice which was either good or poor. Independent variables were socio-demographic variables (age, sex, marital status, work experience, profession, and level of education), training on TB/TBIC, working department, knowledge on TBIC, attitude towards TBIC, knowing the availability of guideline and knowing the availability of TBIC plan.

### Operational definitions

- Good practice:—Participants who practiced 'always' more than or equal 80% practice activities (questions) during three consecutive visits [13].
- Good knowledge:—Participants who scored greater than or equal 80% for knowledge statements were considered as having good TBIC knowledge [17].
- Good attitude:—Participants who agreed for greater than or equal 80% of attitude statements considered as having a positive attitude towards TBIC practice [17].

### Data collection procedures (instrument, personnel)

Four diploma nurses and one supervisor were recruited as data collectors; and they were given one-day training. Data were collected using a self-administered structured questionnaire for socio-demographic information, awareness factors, knowledge, and attitude status towards TBIC. Data collectors visited all 6 hospitals and explained the aim of the research to the facility managers and study participants. Data collectors distributed questionnaires to study participants and had collected the completed questionnaire on the same day. The data collectors completed observational checklists of TBIC measures.

### Data quality control

The questionnaires were prepared first in English, then translated to the Amharic language, and then again back-translated to English. The pre-test was done on 5% of the sample at Shele health center and modification of some questions was made before the main data collection. Data were checked daily for completeness and consistency. Data were coded manually, and to assure the quality of data during data entry 10% of the data were re-entered into EpiData Version 4.4.2.1. Then data cleaning was performed by SPSS Version 21 software to check and correct inconsistencies and missing values. For this purpose, frequency and cross-tabs were utilized.

### Data processing and analysis

Data were exported from EpiData Version 4.4.2.1 to SPSS version 21 statistical packages for analysis. Missing values were checked and labeled accordingly. The recoding of variables was done. The median and inter-quartile range was used to describe age; categorical variables were described by frequency counts and percentages. Multicollinearity between the independent variables was checked by the standard error (SE). Model goodness-of-fit was checked by the Hosmer-Lemeshow statistic. Bivariate logistic regression was used to determine the association between the dependent and each independent variable by using the enter method. Those variables with a P-value of 0.25 and below were candidates for multivariable logistic regressions. Using forward stepwise logistic regression the predictors for TBIC practice were identified. Odds ratio (crude and adjusted odds ratio) with their 95% CI were calculated. P-value <0.05 was considered to decide the statistical significance.

### Ethical consideration

The study was approved by the Institutional Research Ethics Review Board Office of the College of Medicine and Health Sciences, Arbaminch University. A formal letter was given to Gamo Gofa Zone Health Office and then a supporting letter was written to hospital administers. Verbal consent was taken from each study participants after briefing the aim of the study.

## Result

### Socio-demographic characteristics of the participants

A total of 411 participants were included in the study. The response rate was 97.4%. The remaining 2.6% were refused to participate. The majority (60.6%) of the participants were males. The median age was 27 years with an inter-quartile range of 5 years (Table 1).

**Table 1:** Socio-demographic characteristics of healthcare workers in Gamo Gofa Zone, Southern Ethiopia, 2019 (n = 411).

Characteristics		Frequency	Percentage
Age	18–29	302	73.5
	30–39	83	20.2
	> = 40	26	6.3
Sex	Male	249	60.6
	Female	162	39.4
Educational level	Diploma	202	49.3
	Degree and above	208	50.7
Current profession	Pharmacy	52	12.7
	Physician	52	12.7
	Health officer	35	8.5
	Nurse	218	53
	Lab technician	46	11.2
	Radiographer	8	1.9

### Healthcare workers’ knowledge and attitude towards TBIC practice

The study showed that more than two-thirds (68.9%) of HCWs had good knowledge of TBIC measures. Two hundred seventy-six (67.2%) HCWs had a good attitude towards TBIC practice. Only 34.9% of HCWs ever had TB-related training.

### Healthcare workers TBIC practice status

**Fig 1:**
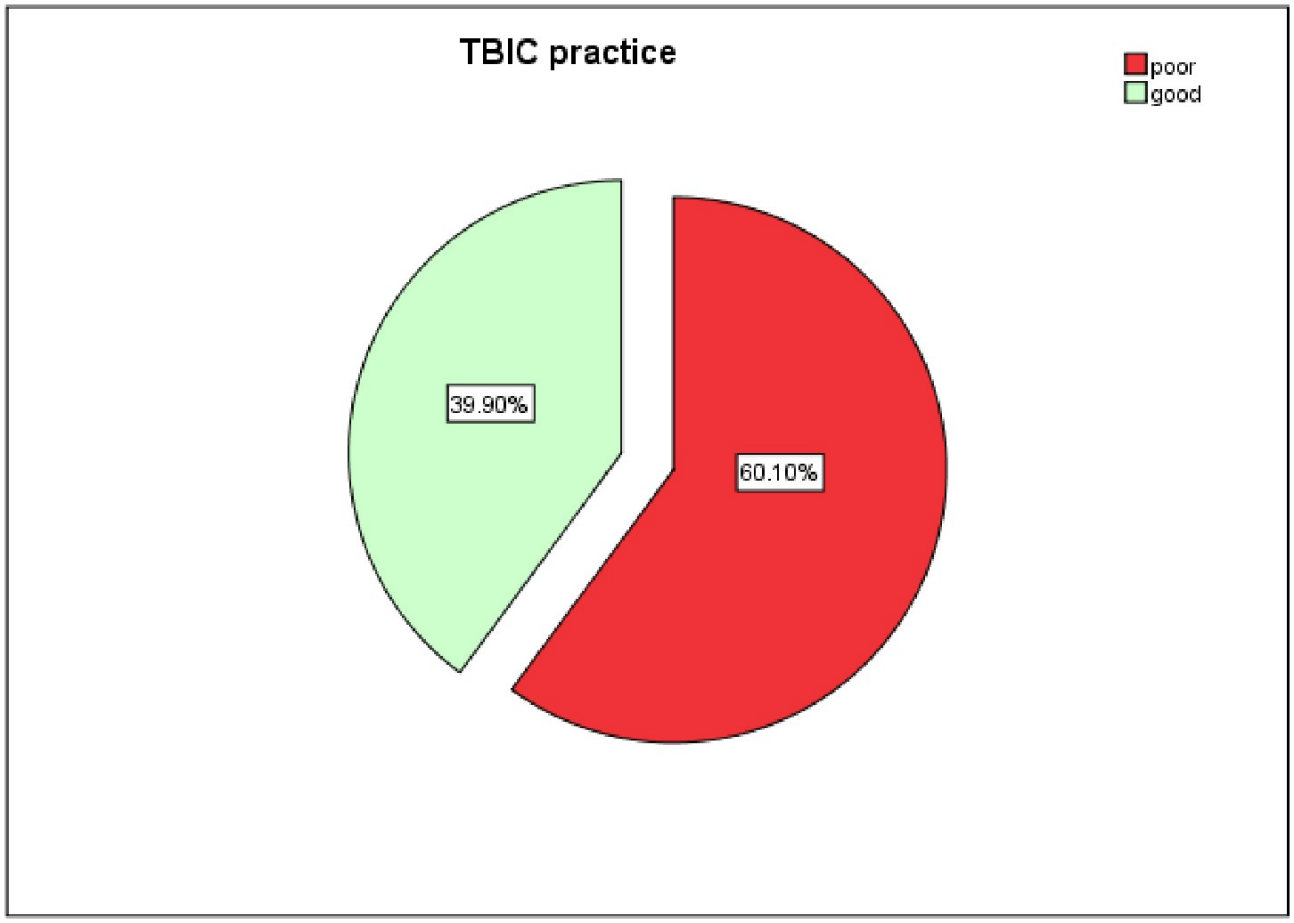
Tuberculosis infection control practice of healthcare workers, in Gamo Gofa Zone, Southern Ethiopia, 2019.

The study showed that the overall good practice among HCWs was 39.9% [95% CI, (35.5, 44.9)] (Fig 1). A total of 247 (60.1%) HCWs scored 80% and above while 164 (39.9%) HCWs had scored below the cut-point (Table 2).

**Table 2:** The distribution of healthcare workers by their practices of TBIC measures in public hospitals; Gamo Gofa Zone, Ethiopia, 2019.

Practice items	Always n (%)	Sometimes n (%)	Never n (%)
HCWs follow TB treatment guideline to treat smear-positive patients	242(58.9)	-	169(41.1)
HCWs open windows when TB presumptive patients are in the room	386(94)	10(2.4)	15(3.6)
Screening tuberculosis for all patients	167(40.6)	7(1.7)	237(57.7)
Giving priority to patients coughing in the waiting area	343(83.5)	7(1.7)	61(14.8)
Giving education for patients how to cough and sneezing	315(76.6)	22(5.4)	74(18)
Use fan	79(69.9)	20(17.7)	14(12.4)
Posters on cough etiquettes displayed on place	163(39.7)	-	248(60.3)
Use AFB as diagnostic tools for TB suspected patients	329(98.2)	-	6(1.8)
Use a respirator/ N95 face-mask/	59(43)	9(6.6)	69(50.4)
Check if a respirator /N95 face-mask/ is airtight and does not allow air leakage	43(63.2)	12(17.6)	13(19.1)
Give face mask for TB patients	105(59)	31(17.4)	42(23.6)
Screen TB patients for HIV	334(98.5)	-	5(1.5)
Practice Status		Good	247(60.1)
		Poor	164(39.9)

Three hundred eighty-six (94%) HCWs opened windows when TB-presumptive patients are in the working rooms. Only 167 (40.6%) HCWs 'always' screened patients for TB and 83.5% of HCWs had given priorities for patients with cough (Table 2).

### Factors associated with TBIC practice

The association of age, sex, marital status, current profession, working department, work experience (service years), knowledge on TBIC, attitude towards TBIC, training on TB/TBIC, the availability of TBIC guideline and TBIC plan in the facility with good TBIC practice were ascertained by using logistic regression.

To identify candidate variables for multivariate logistic regression, the association between each variable with the outcome variable was performed by using bivariate logistic regression. Working unit, current profession, educational level, service year, TB-related training, knowledge on TBIC, attitude towards TBIC, knowing the availability of TBIC plan and guideline were candidates for multivariate logistic regression (Table 3).

**Table 3:** Bivariate and multivariable logistic regression analysis of factors associated with TBIC practice of healthcare workers in Gamo Gofa Zone, Southern Ethiopia, 2019.

Factors	Practice		COR 95% CI	P-value	AOR 95% CI
	Good freq.	Poor freq.			
Educational level					
Degree & above	105	103	2.53 (1.68, 3.81)	0.001*	2.78 (1.70, 4.53)**
Diploma	58	144	1.00		1.00
Current profession					
Physician	31	21	11.32(4.10,31.23)	0.001*	
Health officer	19	16	9.10(3.09, 26.80)	0.001*	
Nurse	79	139	4.36 (1.78,10.66)	0.001*	
Lab. technician	28	18	11.93(4.23,33.62)	0.000*	
Radiographer	1	7	1.09 (0.11, 10.51)	0.937	
Pharmacy	6	46	1.00		
Working unit					
OPD	33	43	5.88 (2.24, 15.43)	0.001*	2.68 (0.95, 7.56)
Emergency	26	62	3.21(1.22, 8.45)	0.018*	2.31 (0.83, 6.45)
Ward	49	47	7.99(3.12, 20.46)	0.001*	4.19(1.53,11.48)**
Laboratory	28	18	11.93 (4.23, 33.62)	0.001*	8.60(2.83, 26.10)**
Triage	4	15	2.04 (0.51, 8.23)	0.314	0.87 (0.19, 3.86)
Radiographer	1	9	0.85 (0.091, 7.96)	0.888	0.54(0.05, 5.49)
TB-clinic	17	7	18.61(5.47,63.33)	0.001*	9.94(2.64,37.41)**
Pharmacy	6	46	1.00		1.00
Service year					
3–5	35	57	0.82(0.49, 1.38)	0.456	
5–10	38	71	0.72(0.44,1.18)	0.187	
>10	17	20	1.14 (0.56, 2.32)	0.724	
< = 3	74	99	1.00		
Training					
Yes	84	59	3.33(2.18, 5.08)	0.001*	2.02(1.24,3.31)**
No	80	187	1.00		1.00
Knowledge					
Good	140	143	4.24 (2.57, 7.00)	0.001*	3.65(2.07,6.43)**
Poor	24	104	1.00		1.00
Knowing the availability of TBIC plan					
Yes	142	193	1.81 (1.05, 3.10)	0.032*	
No	22	54	1.00		
Knowing the availability of TBIC guideline					
Yes	151	203	2.52(1.31, 4.84)	0.006*	
No	13	44	1.00		
Attitude					
Good	123	153	1.84 (1.19, 2.58)	0.006*	
Poor	41	94	1.00		

* = variables candidate for multivariate logistic regression

** = variables significantly associated with TBIC practice at multivariate logistic regression using stepwise forward LR method

freq. = frequency; COR = Crude Odds Ratio; AOR = adjusted Odds Ratio

In identifying independent factors for good TBIC practice, a statistical association was observed among HCWs whose educational level was a degree and above [AOR = 2.78, 95% CI (1.70, 4.53)] in comparison to diploma holders. Those HCWs who had good knowledge about TBIC were three times more likely to have good TBIC practice [AOR = 3.65, 95% CI (2.07, 6.43)]. Furthermore, the difference in the working unit also observed; HCWs working at TB-clinic [AOR = 9.94, 95% CI (2.64, 37.41)] were 9 times more likely to practice TBIC. Participants working at laboratory [AOR = 8.60, 95% CI (2.83, 26.10)] were eight times more likely to practice TBIC while those working at ward [AOR = 4.19, 95% CI (1.53, 11.48)] were four times more likely to have good TBIC practice.

Attitudes, the current profession of HCWs, knowing the availability of TBIC plans and guidelines in the facility were significantly associated with TBIC practice during bivariate logistic regression analysis. However, they did not remain as predictors during multivariate logistic analyses.

## Discussion

Tuberculosis infection control practice among healthcare workers in hospitals of Gamo Gofa Zone was 39.9% [95% CI, (35.5, 45)]. Educational status, working unit, ever having TB-related training, and knowledge about TBIC were identified as predictors for good TBIC practice.

In this study, the overall good TBIC practice was consistent with the finding from West Gojjam (38%) [13]. However, it was lower compared to findings from North-west Ethiopia (63.2%), Addis Ababa (51.4%), and South Africa (72.9%) [14, 17, 19]. The discrepancy might be due to the difference in methodological approaches. These studies used self-report as a means of data collection for practice assessment, and a higher percentage of good TBIC practice might be due to desirability bias. The health facility setups and attitudes of HCWs in South Africa might be better compared to Ethiopia, and this might enables HCWs to practice TBIC measures.

It was found that the odds of TBIC practice were three times more likely among the educational level of degree and above than diploma holders. This was inconsistent with findings from Addis Ababa (AOR = 0.64) [19]. In this study, among HCWs whose educational level of degree and above, 72.1% of them had good knowledge. However, only 65.3% of diploma holders had good knowledge. This study showed also those who had good knowledge had more likely to practice than their counterparts. Also, this might be explained by those degree holders got more knowledge during their academic years and might be due to variation in visiting the different source.

Health care workers working in the laboratory department were eight times more likely to practice TBIC compared to those working in the pharmacy department. This might be due to laboratory personnel take different samples from patients, and to be safe they had practiced infection control measures relative to the pharmacy department. Participants who worked at wards or working at TB-clinic were more likely to practice TBIC than those working in the pharmacy department. This was consistent with findings from West Gojjam Zone (AOR = 2.74 at wards, AOR = 10.17 at TB-clinic) Ethiopia [13]. This can be due to TB patients linked to TB-clinics and wards after diagnosis, and this might increase practicing of the available control measures to protect themselves from TB. This might be also revealed that TB infection was not the concern of all working units, and the task of TBIC was left to those working in TB-clinic, wards, and laboratory despite the possibility that OPD, emergency, and radiographic unit are risky departments.

Healthcare workers who had TB-related training were two times more likely to practice TBIC measures than those who had not the training. This was consistent with findings from Addis Ababa (AOR = 1.48), Ethiopia [19]. Mostly training is practical-aided and provided by experts. This might raise the awareness of healthcare providers to practice TBIC measures appropriately. For instance, in this study, among HCWs who had TB-related training, 62.7% 'always' used N-95 respirator but only 31.8% of HCWs used it among those who had no training. Among N-95 respirator users, 76.5% of trained HCWs used the mask appropriately ('always' checked respirator-fit test) but only 50% of HCWs checked fit tests among those who had no training.

Participants who had good knowledge of TBIC were three times more likely to practice TBIC than those who had poor knowledge. This was in line with findings from South Africa (AOR = 4.030)] This can be explained by having enough knowledge about tuberculosis infection control measures leads to health care workers to practice infection control measures that enable them to protect themselves and patients from tuberculosis.

In previous studies knowing the availability of TBIC plans and guidelines in the health facilities, work experience and attitudes had a significant association with good TBIC practice. However, they had no significant association in this study.

### Strength and limitation of the study

Since practice assessed by observation it gives better information than self-reported practice assessed studies. Observer bias, fail to assess patient-related factors, fail to study the effect of variation in hospital-level (district or general) on practice, and lack of references, because many pieces of research were descriptive cross-section study, were a limitation of this study.

## Conclusion and recommendation

### Conclusion

The overall TBIC practice among healthcare workers in hospitals of Gamo Gofa Zone was 39.9% [95% CI, (35.5, 45)]. The tuberculosis infection control practice of healthcare workers was determined by their educational level, working department, knowledge on tuberculosis infection control practice, and having TB-related training.

### Recommendation

For Regional, Zonal Health office and NGOs

- Participate in providing training for healthcare workers
- Upgrading educational level should also target diploma holders

For health facilities

- Give training for healthcare workers

Healthcare workers

- Healthcare workers should develop their knowledge of TBIC measures.

For researchers

- Researchers better to do qualitative studies that can further explore the reason for not practicing infection control practices measures.

## Supporting information

S1 Fig (TIF)

S1 Table Calculated sample size for the second specific objective using two populations proportions by Open Epi version7. (DOCX)

S1 File (DOCX)

S1 Data (SAV)
