# Supplementary figures and images for: Tuberculosis infection control practices and associated factors among healthcare workers in hospitals of Gamo Gofa Zone, Southern Ethiopia, institution-based cross-sectional study
Source: PLoS One. 2020 Sep 21;15(9):e0239159. doi: 10.1371/journal.pone.0239159 (PMC7505450; doi:10.1371/journal.pone.0239159)

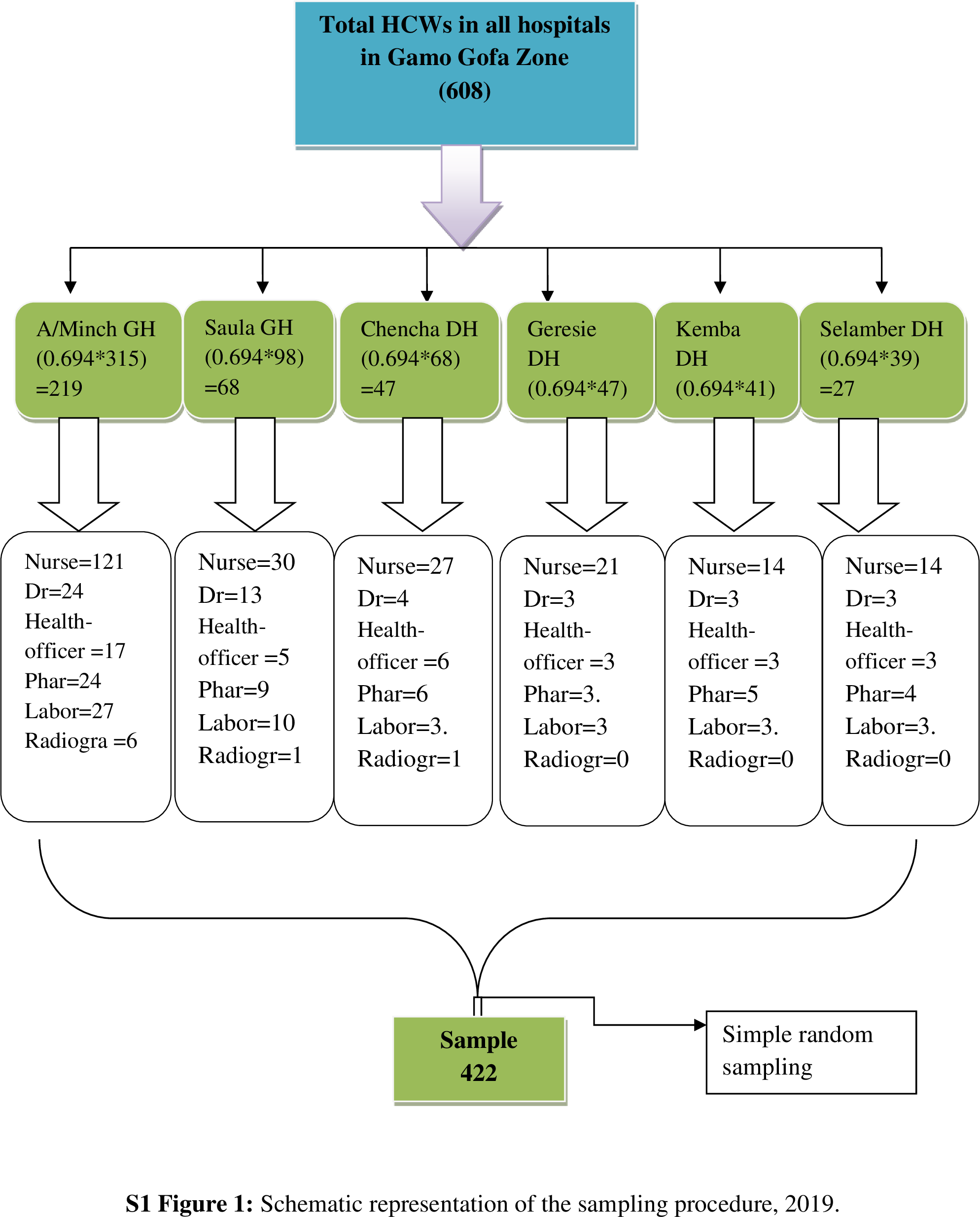

Supplement: S1 Fig — (TIF) [file pone.0239159.s001.tif]
