# Supplementary material for: Tuberculosis infection control practices and associated factors among healthcare workers in hospitals of Gamo Gofa Zone, Southern Ethiopia, institution-based cross-sectional study
Source: PLoS One. 2020 Sep 21;15(9):e0239159. doi: 10.1371/journal.pone.0239159 (PMC7505450; doi:10.1371/journal.pone.0239159)
Supplement: S1 Table — (DOCX) [file pone.0239159.s002.docx]

**S1 Table 1:** Calculated sample size for the second specific objective using two populations proportions by Open Epi version7

| Assumptions | Variables | | |
| --- | --- | --- | --- |
|  | having TB training | Know the presence of TBIC plan | Know the presence of national guideline for TBIC |
|  | - Two-sided CL=95% - Power = 80% - Ratio of Unexposed to Exposed = 1 - % of unexposed with outcome = 45.4% - OR =1.48 | - Two-sided CL=95% - Power = 80% - Ratio of Unexposed to Exposed = 1 - % of unexposed with outcome = 11.1% - OR=8.95 | - Two-sided CL=95% - Power = 80% - Ratio of Unexposed to Exposed = 1 - % of unexposed with outcome = 5.9% - OR=4.25 |
| Calculated n | 862 | 46 | 184 |
| After using correction formula for finite pop. | 862/(1+(862/913))  =443 |  |  |
| Final n (10%  contingency) | 487 | 50 | 202 |
