# Supplementary material for: Tuberculosis infection control practices and associated factors among healthcare workers in hospitals of Gamo Gofa Zone, Southern Ethiopia, institution-based cross-sectional study
Source: PLoS One. 2020 Sep 21;15(9):e0239159. doi: 10.1371/journal.pone.0239159 (PMC7505450; doi:10.1371/journal.pone.0239159)
Supplement: S1 File — (DOCX) [file pone.0239159.s003.docx]

## Information Sheet

ARBA MINCH UNIVERSITY

COLLEGE OF MEDICINE AND HEALTH SCIENCE

DEPARTMENT OF PUBLIC HEALTH

Good morning/afternoon dear participant! My name is _________________. I am working as a data collector for the study being conducted in this institution on Tuberculosis Infection Control Practices and Associated Factors among Healthcare Workers in Hospitals of Gamo Gofa Zone, Southern Ethiopia by Addisu Walelign, who is studying for his master’s degree at Arba Minch University, college of Medicine and Health sciences. I kindly request you to lend me your attention to explain about the study and how being you selected as the study participant.

**Title of the Research Project:** tuberculosis infection control practices and associated factors among healthcare workers in hospitals of Gamo Gofa Zone, Southern Ethiopia

**Name of the Sponsor**: Self

**Purpose of the Research Project**: The aim of the study is to determine the level of tuberculosis infection control practices and to identify factors associated with tuberculosis infection control practices among healthcare workers at hospitals of Gamo Gofa Zone.

**Procedure:** For this study, all hospitals in Gamo Gofa Zone are included. Self-administered questioners are provided to participants and they will answer to questions; then, the data will be collected by the assigned data collectors.

**Risk and Benefits:** The study has no direct benefit for those study participants but they may be indirectly beneficial if the result utilized by planners. Gamo Gofa Zone Health Department, each District Health Office and hospitals will get the final result of the study. There is no a risk due to participating in this study.

.

**Confidentiality**: No need of registering your name; therefore, the information you gave will be kept confidential. All information collected will be kept confidential and destroyed two years after the end of the project. No other person besides the research team will see it.

**Right to Refusal or Withdraw**: To start data collection, approval of the individual participant is required. If you are not willing to participate you can refuse.

**Person to contact**: If you have any further questions or would like to receive further information about the project, please contact:

Name: Addisu Walelign (Principal Investigator)

Dr. Amsalu Alagaw

Mekidim Kassa

Cell Phone: +251 924462429 (Principal Investigator)

Email: [addisuwalelign21@gmail.com](mailto:addisuwalelign21@gmail.com) (Principal Investigator)

[amsalu.alagaw@yahoo.com](mailto:amsalu.alagaw@yahoo.com) (Advisor)

[mekidimka@gmail.com](mailto:mekidimka@gmail.com) (Advisor)

Thank you for taking the time to read the Information Sheet, and asking any questions that you might have had.

## Appendix 2.Questionnaire

**1. Socio-demographic questions**

|  | Questions | Response |
| --- | --- | --- |
| 101 | Age (in year) |  |
| 102 | Sex | 1. Male 2. Female |
| 103 | What is your current educational level? | 1. Diploma 2. Degree and above |
| 104 | Marital status | 1. Single 2. Married 3. Divorced 4. Widowed |
| 105 | What is your current profession? | 1. Physician 2. Health officer 3. Nurse 4. Pharmacy 5. Lab. Technician 6. Radiographer |
| 106 | Where is your current working unit? | 1. OPD 2. Ward 3. Laboratory 4. Pharmacy 5. Radiographic unit 6. Emergency units 7. Triage 8. TB clinic |
| 107 | Years of services | ……… |
| 108 | Have you ever trained for TB/TBIC? | 1. Yes 2. No |

**2. Questions assessing knowledge towards TBIC**

Answer the following statements as ‘Yes’ or ‘No’.

|  | Questions | Response | Remark |
| --- | --- | --- | --- |
| 201 | The doors and windows of a room should be left open. | 1. Yes 2. No |  |
| 202 | TB presumptive patients should be separated from other patients. | 1. Yes 2. No |  |
| 203 | HCWs should minimize the time TB patients spend in health facilities. | 1. Yes 2. No |  |
| 204 | A surgical mask cannot protect the HCWs from TB. | 1. Yes 2. No |  |
| 205 | The respirator /N95 face-mask/ protect the HCWs from TB. | 1. Yes 2. No |  |
| 206 | TB patients have to be counseled to cover their mouth with a handkerchief. | 1. Yes 2. No |  |
| 207 | Every facility should establish an infection prevention committee. | 1. Yes 2. No |  |
| 208 | TB presumptive patients should get priority. | 1. Yes 2. No |  |
| 209 | Regular screening of health workers for TB is one of the TBIC measures. | 1. Yes 2. No |  |
| 210 | Fans can be used to reduce TB transmission in TB ward. | 1. Yes 2. No |  |
| 211 | Respirator /N95 face-mask/ required a fit check. | 1. Yes 2. No |  |
| **Questions assessing awareness of Health care workers** | | | |
| 212 | Is TBIC guideline available in your facility? | 1. Yes 2. No |  |
| 213 | Is TBIC plan available in your facility? | 1. Yes 2. No |  |

**3. Questions assessing the attitude of HCWs towards TBIC**

Answer the following statements as ‘Agree’, ‘Disagree’ or ‘Neutral’. (Circle one)

|  | Questions | Response | Remark |
| --- | --- | --- | --- |
| 301 | There is a need for guidelines regarding TBIC in a health care facility. | 1. Agree 2. Disagree 3. Neutral |  |
| 302 | Health care workers should wear respirators /N95 face-mask/ while caring for TB patients. | 1. Agree 2. Disagree 3. Neutral |  |
| 303 | Respirators/N95 face-mask/ can protect against drug-resistant TB. | 1. Agree 2. Disagree 3. Neutral |  |
| 304 | Even after a patient with TB leaves the room I am working in, I remain at risk of contracting TB. | 1. Agree 2. Disagree 3. Neutral |  |
| 305 | Although most Healthcare workers are already infected there is a need for infection control measures. | 1. Agree 2. Disagree 3. Neutral |  |
| 306 | I wear a respirator/N95 face-mask/ although my patients do not like me to wear it. | 1. Agree 2. Disagree 3. Neutral |  |
| 307 | I am concerned about being infected with TB. | 1. Agree 2. Disagree 3. Neutral |  |
| 308 | There is a need to screen HCWs who might be exposed to tuberculosis. | 1. Agree 2. Disagree 3. Neutral |  |
| 309 | I may not turn off fans if they become noisy or cause cold air. | 1. Agree 2. Disagree 3. Neutral |  |
| 310 | Sputum induction procedures in wards put health care workers at an increased risk of getting infected with TB. | 1. Agree 2. Disagree 3. Neutral |  |
| 311 | Cough hygiene has a role to play in infection control. | 1. Agree 2. Disagree 3. Neutral |  |

**4. Questions assessing practice towards TBIC**

Answer the following statements as ‘Always’, ‘Sometimes’ and ‘Never’. (Circle one)

|  | Questions | Response | Remark |
| --- | --- | --- | --- |
| 401 | Is TB treatment available in your working class? | 1. Yes 2. No | Check the availability if the answer is yes |
| 402 | Do health care workers open windows when coughing/TB presumptive patients are in the room? | 1. Always 2. Sometimes 3. Never |  |
| 403 | Do health care workers screen patients for tuberculosis at their working unit? | 1. Always 2. Sometimes 3. Never |  |
| 404 | Do health care workers give priority to patients coughing in the waiting area? | 1. Always 2. Sometimes 3. Never |  |
| 405 | Do health care workers give counseling for TB presumptive patients how to cough and sneezing? | 1. Always 2. Sometimes 3. Never |  |
| 406 | Is fan available in the working class? | 1. Yes  2. No | If the answer is No, skip to question 408 |
| 407 | If Yes for question 4.06, do health care workers use fans? | 1. Always 2. Sometimes 3. Never |  |
| 408 | Are posters on cough etiquettes are displayed in the required place? | 1. Yes  2. No | Check the availability if the answer is yes |
| 409 | Do HCWs use AFB as diagnostic tools for TB suspected patients? | 1. Always 2. Sometimes 3. Never |  |
| 410 | Is respirator /N-95 facemask/ available in the facility? | 1. Yes  2. No | If the answer is No, skip to question number 413 |
| 411 | If the answer is Yes for question 4.10, do health care workers use a respirator/ N9-5 face-mask/? | 1. Always 2. Sometimes 3. Never | If the answer is Never, skip to question number 413 |
| 412 | If the answer is Yes for question 4.11, do HCWs check if a respirator /N-95 face-mask/ is airtight and does not allow air leakage? | 1. Always 2. Sometimes 3. Never |  |
| 413 | Is face mask available for TB patients? | 1. Yes  2. No | If the answer is No, skip to question 415 |
| 414 | If the answer is Yes for question 4.13, do the HCWs give face-mask for TB patients? | 1. Always 2. Sometimes 3. Never |  |
| 415 | Do health care workers screen TB patients for HIV? | 1. Always 2. Sometimes 3. Never |  |

## መጠይቅ

**አርባምንጭ ዩንቨርስቲ**

**የህክምናና ጤና ሳይንስ ኮሌጅ**

**የሕብረተሰብ ጤና ትምህርት ክፍል**

መግቢያ: ሰላም ውድ ተሳታፊያችን! ስሜ ………………………………….እባላለሁ፡፡ በአርባምንጭ ዩንቨርሲቲ የህክምናና ጤና ሳይንስ ኮሌጅ በሕብረተትሰብ ጤና የትምህርት ክፍል ውስጥ የሁለተኛ ድግሪ ተማሪ የሆኑት አዲሱ ዋለልኝ "በጋሞ ጎፋ ዞን ውስጥ በመንግስት ሆስፒታሎች ተቀጥረው በሚሰሩ የጤና ባለሙያዎች የቲቢ (ሳምባ ነቀርሳ) ኢንፌክሽን ቁጥጥር አተገባበር መዳሰስ" በሚል ርዕስ ለሚሰሩት ጥናት መረጃ ሰብሳቢ ነኝ፡፡ በመሆኑም አጠቃላይ ስለጥናቱና እርስዎ እንዴት እንደተመረጡ እንዳብራራለዎት በትህትና እጠይቃለው፡፡

**የምርምሩ ርዕስ:**

- በጋሞ ጎፋ ዞን ውስጥ በመንግስት ሆስፒታሎች ተቀጥረው በሚሰሩ የጤና ባለሙያዎች የቲቢ (ሳምባ ነቀርሳ) ኢንፌክሽን ቁጥጥር አተገባበር መዳሰስ

**ለምርምር ድጋፍ ያደረገው:** በግል

**የጥናቱ ዓላማ:** የዚህ ጥናት ዋና ዓላማ በጋሞ ጎፋ ዞን ውስጥ በሚገኙ ሆስፒታሎች ተቀጥረው በሚሰሩ የጤና ባለሙያዎች የቲቢ (ሳምባ ነቀርሳ) ኢንፌክሽን ቁጥጥር አተገባበር እና አተገባበሩን የሚወስኑትን ነገሮች መለየት ነው፡፡

**አተገባበር:** ጥናቱ በዞኑ በሚገኙ ሁሉም ሆስፒታሎች ይካሄዳል። መጥይቆቹ በጥናቱ የሚሳተፉ ተሳታፊዎች አንብበው መልስ እንዲሰጡ ከተደረገ በኋላ መጠይቆቹ ይሰበሰባሉ።

**ጥቅሞና ጉዳት**: በጥናቱ ውስጥ የሚካተቱት የጤና ባለሙያዎች ምንም ዓይነት ቀጥተኛ ጥቅም አያገኙም። ነገር ግን ጥናቱ ከተጠናቀቀ በኋላ የጥናቱ ውጤትን መሰረት አድርጎ በሚዘጋጁ እቅዶች በተዘዋዋሪ ተጠቃሚ ሊሆኑ ይችላሉ። የጋሞ ጎፋ ዞን ጤና መምሪያ፣የዲስትሪክት ጽ/ቤቶችና የሆስፒታል ስራ አስኪያጆች የጥናቱ ውጤት እንዲደርሳቸው ይደረጋል።በጥናቱ ተሳታፊ በመሆንዎ የሚጎዱት ነገር አይኖርም፡፡

**ሚስጥራዊነት**: የጥናቱ ተሳታፊዎች ስማቸውን መፃፍ የለባቸውም። ስለዚህ የሚሰጡት መረጃው ሚስጥራዊነቱ የተጠበቀ ነው፡፡ መረጃው በጥንቃቄ የሚያዝ ሲሆን ከሁለት ዓመት በኋላ ይቃጠላል፡፡

**መብት:** የጥናቱ ተሳታፊዎች በጥናቱ ያለመሳተፍ መብት አላቸው፤ እንዲሁም በየትኛው ሰዓት ማቋረጥ ይችላሉ።

ስለምርምሩ ጥያቄ ካለዎት ወይም ተጭማሪ ማብራሪያ ካስፈለገዎት የሚከተሉትን አድራሻዎችን ይጠቀሙ።

አዲሱ ዋለልኝ:- ኢ-ሜይል:addisuwalelign21@gmail.com

ሞባይል ስልክ:+251924462429 (የዋና ተመራማሪ)

ዶ/ር አምሳሉ አላጋው:- [amsalu.alagaw@yahoo.com](mailto:amsalu.alagaw@yahoo.com) (አማካሪ)

መቅድም ካሳ፡- [mekidimka@gmail.com](mailto:mekidimka@gmail.com) (አማካሪ)

ጊዜ ሰጥተው ይህንን የመረጃ ቅጽ ስላነበቡና ስለሚኖረዎ ማንኛውም ጥያቄ በቅድሚያ እናመሰግናለን።

## የመረጃ መሰብሰቢያ ፎርም

1. የማህበራዊና ኢኮኖሚያዊ መረጃዎች (Socio-demographic questions)

|  | ጥያቄዎች | መልስ |
| --- | --- | --- |
| 101 | እድሜ |  |
| 102 | ጾታ | 1. ወንድ 2. ሴት |
| 103 | አሁን ላይ ያሉበት የትምህርት ደረጃ | 1. ዲፕሎማ 2. ድግሪ 3. ማስተርስና ከዚያ በላይ |
| 104 | የጋብቻ ሁኔታ | 1. ያላገባ/ች 2. ያገባ/ች 3. የፈታ/ች 4. ሚስቱ የሞተችበት/ ባሏ የሞተባት |
| 105 | አሁን ላይ የሚሰሩበት የሙያ ዘርፍ ምንድ ነው? | 1. ሐኪም 2. ጤና መኮነን 3. ነርስ 4. ፋርማሲስት 5. ላቦራቶሪ-ቴክኒሰሽያን/ ማይክሮባዮሎጅስት 6. ራድዮግራፈር |
| 106 | አሁን የሚሰሩበት የስራ ክፍል የት ነው? | 1. ተምላላሽ የህክምና ክፍል 2. ድንገተኛ ክፍል 3. የተኝቶ ህክምና ክፍል 4. ላቦራቶሪ 5. ትሪያጅ 6. ጨረራ ክፍል 7. ፋርማሲ 8. ቲቢ ክሊኒክ |
| 107 | የአገልግሎት ዓመት |  |
| 108 | ስለ ቲቢና /TB/ ቲቢን መከላከል በተመለከተ ስልጠና ወሰደው ያውቃሉ? | 1. አዎ  2. አይደለም |

**2. ስለ ቲቢ በሽታ ቁጥጥር ግንዛቤ (እውቀት) የሚዳስሱ ጥያቄዎች (መልሱን ያክብቡ) (Questions assessing knowledge towards tuberculosis infection control practice)**

|  | ጥያቄዎች | መልስ | Remark |
| --- | --- | --- | --- |
| 201 | በስራ ክፍሎች የሚገኙ በሮችና መስኮቶች ክፍት መሆን አለባቸው፡፡ | 1. አዎ  2. አይደለም |  |
| 202 | የቲቢ ተጠርጣሪዎች ከሌሎች ታካሚዎች መለየት አለባቸው፡፡ | 1. አዎ  2. አይደለም |  |
| 203 | የቲቢ ታማሚዎች በጤና ተቋም ውስጥ የሚኖራቸውን የቆይታ ጊዜ ማሳጠር ይገባል፡፡ | 1. አዎ  2. አይደለም |  |
| 204 | ሰርጅካል ማስክ( Surgical mask) ጤና ባለሙያውን በቲቢ ከመያዝ አይከላከልም፡፡ | 1. አዎ  2. አይደለም |  |
| 205 | ሪስፓራተር /N95 face-mask/ መጠቀም የጤና ባለሙያዎች ከቲቢ ሊከላከላል ይችልላል፡፡ | 1. አዎ  2. አይደለም |  |
| 206 | የቲቢ ታማሚዎች አፋቸውን በመሀረብ እንዲሸፍኑ ትምህርት መሰጠት ይኖርበታል፡፡ | 1. አዎ  2. አይደለም |  |
| 207 | ሁሉም የጤና ተቋማት የኢንፌክሽን መከላከልና መቆጣጠር ኮሚቴ ሊኖራቸው ይገባል፡፡ | 1. አዎ  2. አይደለም |  |
| 208 | ለቲቢ ተጠርጣሪዎች ቅድሚያ መስጠት ያስፈልጋል፡፡ | 1. አዎ  2. አይደለም |  |
| 209 | መደበኛ የሆነ የቲቢ ተጋላጭነት ምርመራ ለጤና ባለሙያዎች ማድረግ አንዱ የቲቢ በሽታን መቆጣጠሪያ መንገድ ነው፡፡ | 1. አዎ  2. አይደለም |  |
| 210 | የኤሌክትሪክ ነፋስ (ቬንትሌተር) የቲቢ በሽታን መተላለፍ ለመቀነስ ይረዳል፡፡ | 1. አዎ  2. አይደለም |  |
| 211 | የሪስፓራተር /N95 face-mask/ ተስማሚነትት( fit test) ማረጋገጥ ያስፈልጋል፡፡ | 1. አዎ  2. አይደለም |  |
|  | KNLOWLEDGE STATUS | 1. Good |  |
|  |  | 2. Poor |  |
| **የጤና ባለሙያው በተቋሙ ውስጥ ስለሚገኙ ማቴሪያሎች ግንዛቤ** | | | |
| 212 | በሚሰሩበት ጤና ተቋም የቲቢ ህክምና መምሪያ አለን? | 1. አዎ  2. የለም |  |
| 213 | በሚሰሩበት ጤና ተቋም የቲቢ የኢንፌክሽን  መከላከልና መቆጣጠር ዕቅድ አለን? | 1. አዎ  2. የለም |  |

**3. ስለ ቲቢ በሽታ ቁጥጥር ሁኔታ የሚዳስሱ ጥያቄዎች (አንዱን በመምረጥ ይክበቡ) Questions assessing the attitude of HCWs towards TBIC**

|  | ጥያቄዎች | መልስ | Remark |
| --- | --- | --- | --- |
| 301 | በጤና ተቋማት ውስጥ የቲቢ የኢንፌክሽን መከላከልና መቆጣጠር መምሪያ መኖር አለበት፡፡ | 1. እስማማለው 2. አልስማማም 3. አስተያየት የለኝም |  |
| 302 | የጤና ባለሙያዎች የቲቢ ህክምና አገልግሎት ሲሰጡ N-95 ሪስፓራተር መጠቀም አለባቸው፡፡ | 1. እስማማለው 2. አልስማማም 3. አስተያየት የለኝም |  |
| 303 | ሪስፓራተር /N95 face-mask/ በመጠቀም መድኃኒት ከተላመደ የቲቢ በሽታ መጠበቅ ይቻላል። | 1. እስማማለው 2. አልስማማም 3. አስተያየት የለኝም |  |
| 304 | የቲቢ ታማሚው ከወጣም በኋላ በምሰራበት ክፍል ውስጥ በቲቢ ልያዝ እችላለው። | 1. እስማማለው 2. አልስማማም 3. አስተያየት የለኝም |  |
| 305 | አብዛኛው ባለሙያ በቲቢ ኢንፌክትድ ቢሆንም ኢንፌክሽንን መከላከልና ቁጥጥር ትግበራ ያስፈልጋል፡፡ | 1. እስማማለው 2. አልስማማም 3. አስተያየት የለኝም |  |
| 306 | ታካሚዎች ሪስፓራተር /N95 face-mask/ ስጠቀም ደስ ባይላቸውም እጠቀማለው፡፡ | 1. እስማማለው 2. አልስማማም 3. አስተያየት የለኝም |  |
| 307 | በቲቢ መያዝ ያሳስበኛል፡፡ | 1. እስማማለው 2. አልስማማም 3. አስተያየት የለኝም |  |
| 308 | የቲቢ ተጋላጭነት ምርመራ ለጤና ባለሙያዎች ማድረግ ያስፈልጋል ። | 1. እስማማለው 2. አልስማማም 3. አስተያየት የለኝም |  |
| 3.09 | የኤሌክትሪክ ነፋስ (ቬንትሌተር) ድምፁ ቢረብሽኝም ወይም ቅዝቃዜ ከበዛ እጠቀማለው። | 1. እስማማለው 2. አልስማማም 3. አስተያየት የለኝም |  |
| 310 | ቲቢ ታካሚዎች አክታ በዋርድ ውስጥ እንዲሰጡ ማድረግ ጤ ና ባለሙያውን ለቲቢ ሊያጋልጠው ይችላል። | 1. እስማማለው 2. አልስማማም 3. አስተያየት የለኝም |  |
| 311 | ታካሚው በሚያስልበት ጊዜ አፉን እንዲሸፍን ማድረግ የቲቢ ኢንፌክሽንን ለመከላከል ሚና አለው። | 1. እስማማለው 2. አልስማማም 3. አስተያየት የለኝም |  |

**4. የቲቢ በሽታን ለመቆጣጠር የሚደረጉ ተግባራትን የሚዳስሱ ጥያቄዎች (አንዱን በመምረጥ ይክበቡ) (Questions assessing practice towards TBIC)**

|  | ጥያቄዎች | መልስ | Remark |
| --- | --- | --- | --- |
| 401 | የቲቢ ታማሚዎችን ለማከም የቲቢ ህክምና መመሪያ( guideline) አለ? | 1. አዎ 2. የለም | መኖሩን በምልከታ ያረጋግጡ |
| 402 | የቲቢ ታማሚዎችን በሚያክሙበት ጊዜ መስኮቶችን ይከፍታሉ? | 1. ሁልጊዜ 2. አልፋልፎ 3. በፍፁም |  |
| 403 | በሚሰሩበት ክፍል ውስጥ ለታካሚዎች የቲቢ ልየታ(screening) ያደርጋሉ? | 1. ሁልጊዜ 2. አልፋልፎ 3. በፍፁም |  |
| 404 | ሳል ላለባቸው ታካሚዎች ቅድሚያ ይሰጣሉን? | 1. ሁልጊዜ 2. አልፋልፎ 3. በፍፁም |  |
| 405 | ሳል ላለባቸው ታማሚዎች እንዴት መሳልና ማስነጠስ እንዳለባቸው ትምህርት ይሰጣሉን? | 1. ሁልጊዜ 2. አልፋልፎ 3. በፍፁም |  |
| 406 | የኤሌክትሪክ ነፋስ (ቬንትሌተር-fan) በሚሰሩበትክፍል አለን? | 1. አዎ  2. የለም | መልሰዎ የለም ከሆነ ወደ ጥያቄ 408 ይሻገሩ |
| 407 | ለጥያቄ ቁጥር 406 መልስዎ አዎን ከሆነ የኤሌክትሪክ ነፋስ (ቬንትሌተር-fan) ይጠቀማሉን? | 1. ሁልጊዜ 2. አልፋልፎ 3. በፍፁም |  |
| 408 | ታማሚዎች እንዴት ማሳል እንዳለባቸው የሚያስረዱ ፖስተሮች ሊታይ በሚችል ግልጽ ቦታ ተለጥፎልን? | 1. አዎ 2. የለም | መኖሩን በምልከታ ያረጋግጡ |
| 409 | የቲቢን ተጠርጣሪዎች AFB በመጠቀም ቲቢን ይለያሉ? | 1. ሁልጊዜ 2. አልፋልፎ 3. በፍፁም |  |
| 410 | ሪስፓራተር /N95 face-mask/ በጤና ተቋምዎ ውስጥ ይገኛልን? | 1. አዎ  2. የለም | መልሰዎ የለም ከሆነ ወደ ጥያቄ 413 ይሻገሩ |
| 411 | ለጥያቄ ቁጥር 411 መልስዎ አዎን ከሆነ ባለሙያው ሪስፓይራተር /N-95 face-mask/ ይጠቀማሉን? | 1. ሁልጊዜ 2. አልፋልፎ 3. በፍፁም |  |
| 412 | ሪስፓራተር /N-95 face-mask/ ሲጠቀሙ ተሰማሚነቱን (አየር አለማሰገባቱን፣በቂ መጠን) ያረጋግጣሉን? | 1. ሁልጊዜ 2. አልፋልፎ 3. በፍፁም |  |
| 413 | በተቋምዎ ውስጥ ለቲቢ ታማሚዎች የፊት መሸፈኛ አለን? | 1. አዎ  2. የለም | መልስዎ የለም ከሆነ ወደወጥያቄ 415 ይሻገሩ |
| 414 | ለጥያቄ ቁጥር 413 መልስዎ አዎን ከሆነ የፊት መሸፈኛ ለቲቢ ታማሚዎች ይሰጣሉን? | 1. ሁልጊዜ 2. አልፋልፎ 3. በፍፁም |  |
| 415 | ለቲቢ ታማሚዎች የአች ይ ቪ (HIV) ምርመራ ያደርጋሉን? | 1. ሁልጊዜ 2. አልፋልፎ 3. በፍፁም |  |
